# Eighteen months into the COVID-19 pandemic: The prevalence of depression, anxiety, and stress symptoms in Southeast Asia and the associated demographic factors

**DOI:** 10.3389/fpubh.2022.863323

**Published:** 2022-08-04

**Authors:** Wendy Wan Ying Tay, Jehanita Jesuthasan, Kim Sui Wan, Tiffanie Ong, Feisul Mustapha

**Affiliations:** ^1^Naluri Hidup Sdn Bhd, Kuala Lumpur, Malaysia; ^2^Ministry of Health, Putrajaya, Malaysia

**Keywords:** mental health, COVID-19, anxiety, depression, stress

## Abstract

Mental health has become a growing concern in the wake of the COVID-19 pandemic. We sought to determine the prevalence of mental health symptoms 18 months after the pandemic's declaration. Our cross-sectional study conducted among 18- to 65-year-old adults (*N* = 33,454) in October 2021 using the Depression, Anxiety and Stress Scales (DASS-21) found a high prevalence of severe to extremely severe anxiety (49%), depression (47%) and stress (36%) symptoms in Malaysia, Indonesia, Thailand, and Singapore. Multiple logistic regression showed that female and non-binary genders were associated with increased odds of severe/extremely severe symptoms of anxiety (female: aOR 1.44 [95% CI 1.37–1.52]; non-binary aOR 1.46 [1.16–1.84]), depression (female: aOR 1.39 [1.32–1.47]; non-binary aOR 1.42 [1.13–1.79]), and stress (female: aOR 1.48 [CI 1.40–1.57]; non-binary aOR 1.42 [1.12–1.78]). In all three symptom domains, the odds of severe/extremely severe symptoms decreased across age groups. Middle- and high-income respondents had lower odds of reporting severe/extremely severe anxiety (middle-income: aOR 0.79 [0.75–0.84]; high-income aOR 0.77 [0.69–0.86]) and depression (middle-income: aOR 0.85 [0.80–0.90]; high-income aOR 0.84 [0.76–0.94]) symptoms compared to low-income respondents, while only middle-income respondents had lower odds of experiencing severe/extremely severe stress symptoms (aOR 0.89 [0.84–0.95]). Compared to residents of Malaysia, residents of Indonesia were more likely to experience severe/extremely severe anxiety symptoms (aOR 1.08 [1.03–1.15]) but less likely to experience depression (aOR 0.69 [0.65–0.73]) or stress symptoms (aOR 0.92 [0.87–0.97]). Respondents living in Singapore had increased odds of reporting severe/extremely severe depression symptoms (aOR 1.33 [1.16–1.52]), while respondents residing in Thailand were more likely to experience severe/extremely severe stress symptoms (aOR 1.46 [1.37–1.55]). This study provides insights into the impacts of the COVID-19 pandemic on the point prevalence of psychological distress in Southeast Asia one and a half years after the beginning of the pandemic.

## Introduction

Mental health is a growing concern around the world. In the wake of the COVID-19 pandemic, significant concerns about its impact on mental illness have been raised. Social distancing measures, designed to limit the spread of the virus, and their accompanying impact on social support systems, can contribute to increased depression and anxiety. Moreover, psychological distress can also emerge from fears of infection and loss of employment resulting from economic instability (1).

During the pandemic, screen time increased dramatically, owing to reduced opportunities for face-to-face interaction and offline activities. These changes are likely to have also had a significant effect on mental health, given the association between internet usage and both depression and anxiety (2). Social media use in particular has been found to increase psychological distress. For example, high levels of social media usage, including addictive and compulsive use, and using large numbers of social media sites, can trigger social media fatigue, and, in turn, anxiety and depression (3–6). Moreover, high exposure to COVID-19 information online was shown to have a detrimental impact on mental health, particularly on anxiety symptoms (7–9).

In Southeast Asia during the COVID-19 pandemic, prevalence rates of anxiety and depression have been reported to be 31% and 16% for anxiety and depression, respectively, among the general population (10). Crucially, rates may be even higher among internet users specifically, for the reasons outlined above. Indeed, rates of problematic mental health symptoms in an Australian internet-based sample in March to April 2020 were especially high, at 79% (11). This highlights the importance of examining the prevalence of psychological distress among internet users.

Given the widespread impact of the pandemic on mental health, it is also crucial to identify the groups most affected. This can enable the development and delivery of support tailored to these individuals, in an effort to move toward precision public health. Studies have demonstrated that the impacts of the pandemic have not been equal across demographic groups. Wang et al.'s (12) study on the general population of seven Asian countries found that depression, anxiety, and stress scores varied between countries, age groups, genders, and education backgrounds. However, much of the research describing rates of psychological distress during the COVID-19 pandemic was conducted in the immediate months after the pandemic was announced (13, 14). The public health situation is perpetually evolving with new waves of outbreaks, changing social and movement restrictions, and increasing vaccination coverage. Consequently, the mental health status of the population should continue to be monitored to understand how the mental health impact of the pandemic is changing.

Our study therefore aims to determine the point prevalence of depression, anxiety, and stress symptoms in a Southeast Asian internet-based sample 18 months after the declaration of COVID-19 as a global pandemic and identify the factors associated with severe to extremely severe levels of these symptoms.

## Method

### Design and participants

This cross-sectional study was conducted in October 2021 using an online survey distributed to individuals in four countries in Southeast Asia, namely Malaysia, Indonesia, Singapore, and Thailand. Respondents were working-age adults (18- to 65-years-old) recruited through paid advertisements on social media platforms (Instagram and Facebook) and on Google Search and Google Display to complete the online survey on Naluri's website. There were no tokens or services provided for their participation in this study. Naluri is a Southeast Asian digital health company providing structured multidisciplinary health coaching to support and improve physical and mental health. Respondents who were outside of the target age range, lived outside of the four target countries, or did not answer all demographic questions were excluded.

Ethics approval was obtained from the Sunway Research Ethics Committee (ID 014/2021/IND/ER). All respondents provided digital informed consent and no personally identifiable information was collected.

### Measures and instruments

The survey was composed of two parts: a demographics questionnaire and the 21-item Depression, Anxiety, and Stress Scales (DASS-21) (15). The demographics questionnaire asked respondents to report their gender, year of birth, country of residence, and household income. Three domains of respondent's mental health (depression, anxiety, and stress) were measured using the DASS-21. The DASS-21 is a self-report questionnaire that includes three scales corresponding to the depression, anxiety, and stress domains of mental health. The depression scale assesses anhedonia, hopelessness, low energy, and dysphoria. The anxiety scale refers to autonomic arousal, including agitation and physiological symptoms. The stress scale measures chronic arousal, which entails irritability, tension, and nervousness. Each scale contains seven items, which the respondents score on a scale of 0 (did not apply to me at all) to 3 (applied to me very much, or most of the time). The items for each subscale are summed and multiplied by a factor of two, yielding a score ranging 0 to 42 for each subscale. These scores can be categorized into five categories, namely normal, mild, moderate, severe, and extremely severe, using the cut-offs proposed by Lovibond and Lovibond (15). The primary outcome in the current study was the prevalence of severe/extremely severe depression (score ≥21), anxiety (score ≥15), and stress (score ≥26). We used severe/extremely severe symptoms as the cut-off for regression analyses as the identification of factors associated with this specific group of populations would allow targeted public health interventions.

The DASS-21 has been validated among Asians in previous research (16–19). To minimize reporting bias, the questions were presented in English, Malay, Chinese, Indonesian, or Thai using published translations based on the participant's preference. The Malay, Chinese, and Indonesia versions have been previously validated (19–21), while only the 42-item version of the DASS has been validated in Thai (22). The DASS-21 is in the public domain, so permission is not required to use it.

Respondent's demographic characteristics were age, gender, country of residence, and household income. The response options for gender were male, female, non-binary, and prefer not to answer. Non-binary gender refers to individuals who identify as neither male nor female. Age was used as a 4-level categorical variable using the categories 18–29, 30–39, 40–49, and 50–65. Household income was also used as a categorical variable, using the categories low, middle, and high income. Low income was defined as ≤ MYR 5,000, ≤ IDR 5,000,000, ≤ SGD 2,000, and ≤ THB 15,000 for residents of Malaysia, Indonesia, Singapore, and Thailand, respectively. High income was defined as > MYR 11,000, > IDR 12,000,000, > SGD 18,000, and > THB 50,000 for residents of Malaysia, Indonesia, Singapore, and Thailand, respectively. Respondents reporting incomes between these cut-offs were categorized as middle-income. For Singapore and Malaysia, household income level thresholds were defined based on government definitions (23, 24). For Indonesia and Thailand, middle income was defined as household income between the 20th to 80th percentile of the income distribution (25). Low income and age 18 to 29 years were chosen as the reference categories as these made up the largest portion of the sample. Malaysia was chosen as the reference category for country of residence as the study was designed and conducted by researchers based in Malaysia.

### Data analyses

Data for depression, anxiety, and depression scores are presented in mean ± standard deviation (SD). Both frequency and percentages are reported for categorical variables. The 95% confidence intervals are also presented for the prevalence of depression, anxiety, and stress symptoms.

Simple logistic regressions were performed to examine the influence of each of the independent variables on the odds of experiencing severe to extremely severe symptoms of depression, anxiety, and stress. Variables with *p* < 0.25 were included in the multiple logistic regression model using forward likelihood ratio. The Omnibus test of model coefficients of determination, *R*^2^, Hosmer & Lemeshow, classification table, and area under the Receiver Operating Characteristic (ROC) curve were reported. Data analyses were performed using statistical software (*R* version 4.02).

## Results

### Participant characteristics

Responses from 33,454 respondents who met the inclusion criteria were analyzed. The median age of our study population was 23 years (interquartile range 8). Sample characteristics are reported in Table 1. The majority of the sample was female (75.96%), 18- to 29-years-old (72.53%), and low-income (73.41%).

**Table 1:** Respondent characteristics and mean depression, anxiety, and stress scores.

		**N**	**%**	**Mean anxiety score (SD)**	**Mean depression score (SD)**	**Mean stress score (SD)**
**Gender**	Male	7,726	23.09%	14.11 (9.71)	18.03 (11.94)	18.72 (10.57)
	Female	25,411	75.96%	16.42 (10.23)	20.67 (12.09)	21.31 (10.64)
	Other	317	0.95%	16.79 (9.68)	22.18 (10.62)	23.34 (9.29)
**Age**	18–29	24,264	72.53%	17.16 (9.97)	21.46 (11.72)	21.94 (10.28)
	30–39	6,701	20.03%	13.35 (9.85)	17.49 (12.28)	18.49 (10.95)
	40–49	2,001	5.98%	10.91 (9.73)	14.03 (12.00)	15.62 (10.98)
	50–65	488	1.46%	8.01 (8.01)	11.10 (10.99)	12.37 (9.96)
**Country**	Malaysia	10,319	30.85%	15.34 (10.72)	20.49 (12.85)	19.38 (11.27)
	Indonesia	12,590	37.63%	16.87 (10.03)	19.02 (12.07)	20.46 (10.45)
	Singapore	1,063	3.18%	15.01 (10.24)	21.45 (11.95)	19.96 (10.05)
	Thailand	9,482	28.34%	15.29 (9.56)	20.85 (11.16)	22.65 (10.08)
**Income**	Low	24,559	73.41%	16.61 (10.20)	20.60 (12.10)	20.99 (10.66)
	Middle	7,181	21.47%	14.08 (9.72)	18.80 (11.88)	20.08 (10.61)
	High	1,714	5.12%	13.17 (9.76)	17.81 (12.23)	19.86 (10.98)
**Total sample**		33,454	100.00%	15.89 (10.15)	20.07 (12.10)	20.73 (10.67)

### Prevalence of psychological distress

The prevalence of anxiety, depression, and stress symptoms for each level of severity in each country and across the sample is shown in Table 2. In our sample, 46.86% had severe to extremely severe symptoms of depression. Anxiety symptoms were experienced at a rate of 49.34%, while 36.19% of the sample had severe or above stress symptoms. In addition, 61.24% of the sample had severe or above symptoms in at least one of the three domains.

**Table 2:** Prevalence of depression, anxiety, and stress symptoms at each level of symptom severity in each country and across the sample.

		**Anxiety**		**Depression**		**Stress**	
		**N**	**% (95% CI)**	**N**	**% (95% CI)**	**N**	**% (95% CI)**
Malaysia	**Normal**	2,889	28.00 (27.13–28.88)	2,481	24.04 (23.22–24.88)	3,939	38.17 (37.23–39.12)
	**Mild**	615	5.96 (5.51–6.44)	948	9.19 (8.64–9.76)	1,119	10.84 (10.25–11.46)
	**Moderate**	1,905	18.46 (17.72–19.23)	1,904	18.45 (17.71–19.22)	1,796	17.40 (16.68–18.15)
	**Severe**	1,216	11.78 (11.17–12.43)	1,402	13.59 (12.93–14.27)	2,062	19.98 (19.22–20.77)
	**Extremely severe**	3,694	35.80 (34.87–36.73)	3,584	34.73 (33.81–35.66)	1,403	13.60 (12.94–14.28)
Indonesia	**Normal**	2,311	18.36 (17.69–19.05)	3,219	25.57 (24.81–26.34)	4,267	33.89 (33.07–34.73)
	**Mild**	884	7.02 (6.58–7.49)	1,344	10.68 (10.14–11.23)	1,649	13.10 (12.52–13.70)
	**Moderate**	2,711	21.53 (20.82–22.26)	2,607	20.71 (20.00–21.43)	2,408	19.13 (18.44–19.83)
	**Severe**	1,696	13.47 (12.88–14.08)	1,850	14.69 (14.08–15.33)	2,494	19.81 (19.12–20.52)
	**Extremely severe**	4,988	39.62 (38.76–40.48)	3,570	28.36 (27.57–29.15)	1,772	14.07 (13.47–14.70)
Singapore	**Normal**	281	26.43 (23.83–29.22)	199	18.72 (16.45–21.23)	370	34.81 (31.96–37.77)
	**Mild**	77	7.24 (5.79–9.01)	89	8.37 (6.81–10.24)	144	13.55 (11.58–15.79)
	**Moderate**	229	21.54 (19.13–24.16)	239	22.48 (20.03–25.14)	217	20.41 (18.05–22.99)
	**Severe**	128	12.04 (10.18–14.19)	164	15.43 (13.34–17.77)	211	19.85 (17.52–22.40)
	**Extremely severe**	348	32.74 (29.94–35.66)	372	35.00 (32.14–37.96)	121	11.38 (9.57–13.48)
Thailand	**Normal**	2,094	22.08 (21.26–22.94)	1,674	17.65 (16.9.0–18.44)	2,353	24.82 (23.95–25.70)
	**Mild**	777	8.19 (7.65–8.77)	975	10.28 (9.68–10.92)	1,173	12.37 (11.72–13.05)
	**Moderate**	2,174	22.93 (22.09–23.79)	2,098	22.13 (21.3–22.98)	1,912	20.16 (19.36–20.99)
	**Severe**	1,293	13.64 (12.96–14.35)	1,648	17.38 (16.63–18.16)	2,370	24.99 (24.13–25.88)
	**Extremely severe**	3,144	33.16 (32.21–34.12)	3,087	32.56 (31.62–33.51)	1,674	17.65 (16.90–18.44)
**Total**	**Normal**	7,575	22.64 (22.20–23.1)	7,573	22.64 (22.19–23.09)	10,929	32.67 (32.17–33.17)
	**Mild**	2,353	7.03 (6.76–7.31)	3,356	10.03 (9.71–10.36)	4,085	12.21 (11.86–12.57)
	**Moderate**	7,019	20.98 (20.55–21.42)	6,848	20.47 (20.04–20.91)	6,333	18.93 (18.51–19.36)
	**Severe**	4,333	12.95 (12.6–13.32)	5,064	15.14 (14.76–15.53)	7,137	21.33 (20.90–21.78)
	**Extremely severe**	12,174	36.39 (35.87–36.91)	10,613	31.72 (31.23–32.23)	4,970	14.86 (14.48–15.24)

The prevalence of severe or above anxiety symptoms was highest in the Indonesian sample (53.09%), followed by the Malaysian (47.58%) and Thai (46.80%) samples, while the Singaporean sample had the lowest prevalence (44.78%). Similar proportions of the samples from Singapore, Thailand, and Malaysia reported depression symptoms (50.43, 49.94, and 48.32%, respectively), while a smaller proportion of the respondents from Indonesia experienced these symptoms (43.05%). Rates of stress were higher among respondents from Thailand (42.64%) than among those from Malaysia, Indonesia, or Singapore (33.58, 33.88, and 31.32%, respectively).

### Factors associated with severe/extremely severe psychological symptoms

Simple logistic regressions revealed that all four demographic variables were factors significantly associated with severe to extremely severe symptoms for depression, anxiety, and stress (Supplementary Table 1). All four variables were therefore entered into multiple logistic regression models for the three outcome variables, the results of which are shown in Tables 3–5.

**Table 3:** Factors associated with severe/extremely severe anxiety symptoms.

**Variable**		**(95% CI)**	**Odds ratio**	**95% CI Lower**	**95% CI Upper**	***p***
**Gender**	Male	42.14 (41.04–43.25)	1.00			
	Female	51.49 (50.87–52.11)	1.44	1.37	1.52	<0.001
	Other	52.68 (47.03–58.27)	1.46	1.16	1.84	0.001
**Age**	18–29	54.54 (53.91–55.17)	1.00			
	30–39	38.73 (37.56–39.91)	0.55	0.52	0.58	<0.001
	40–49	29.39 (27.41–31.44)	0.38	0.35	0.42	<0.001
	50–65	18.44 (15.16–22.23)	0.22	0.17	0.28	<0.001
**Country**	Malaysia	47.58 (46.61–48.55)	1.00			
	Indonesia	53.09 (52.21–53.96)	1.08	1.03	1.15	0.003
	Singapore	44.78 (41.77–47.83)	1.11	0.97	1.28	0.113
	Thailand	46.79 (45.79–47.80)	0.96	0.91	1.02	0.226
**Income**	Low	52.31 (51.68–52.93)	1.00			
	Middle	41.80 (40.66–42.96)	0.79	0.75	0.84	<0.001
	High	38.45 (36.14–40.80)	0.77	0.69	0.86	<0.001

*Omnibus test X^2^ = 1409.22, p < 0.001; Nagelkerke R^2^ =5.5%; Hosmer & Lemeshow test X^2^ = 31.36, p < 0.001; Classification table 58.3% correct; Multicollinearity checks indicated no multicollinearity between the associated factors; ROC area = 0.582*.

**Table 4:** Factors associated with severe/extremely severe depression symptoms.

**Variable**		**% (95% CI)**	**Odds ratio**	**95% CI Lower**	**95% CI Upper**	***p***
**Gender**	Male	39.99 (38.9–41.10)	1.00			
	Female	48.86 (48.25–49.48)	1.39	1.32	1.47	<0.001
	Other	53.63 (47.97–59.20)	1.42	1.13	1.79	0.002
**Age**	18–29	51.56 (50.93–52.19)	1.00			
	30–39	37.65 (36.49–38.83)	0.54	0.51	0.58	<0.001
	40–49	27.74 (25.79–29.76)	0.34	0.31	0.38	<0.001
	50–65	18.24 (14.97–22.02)	0.20	0.16	0.25	<0.001
**Country**	Malaysia	48.32 (47.35–49.29)	1.00			
	Indonesia	43.05 (42.18–43.92)	0.69	0.65	0.73	<0.001
	Singapore	50.42 (47.37–53.47)	1.33	1.16	1.52	<0.001
	Thailand	49.94 (48.93–50.95)	1.04	0.98	1.10	0.240
**Income**	Low	48.51 (47.88–49.14)	1.00			
	Middle	42.95 (41.80–44.10)	0.85	0.80	0.90	<0.001
	High	39.61 (37.30–41.98)	0.84	0.76	0.94	0.002

*Omnibus test X^2^ = 1406.13 p < 0.001; Nagelkerke R^2^ = 5.5%; Hosmer & Lemeshow test X^2^ = 23.72, p = 0.003; Classification table 57.7% correct; Multicollinearity checks indicated no multicollinearity between the associated factors; ROC area = 0.609*.

**Table 5:** Factors associated with severe/extremely severe stress symptoms.

**Variable**		**% (95% CI)**	**Odds ratio**	**95% CI Lower**	**95% CI Upper**	***p***
**Gender**	Male	29.26 (28.25–30.30)	1.00			
	Female	38.21 (37.61–38.81)	1.48	1.40	1.57	<0.001
	Other	43.22 (37.72–48.88)	1.42	1.12	1.78	0.003
**Age**	18–29	39.67 (39.06–40.29)	1.00			
	30–39	29.38 (28.30–30.49)	0.64	0.60	0.68	<0.001
	40–49	22.34 (20.54–24.24)	0.44	0.39	0.49	<0.001
	50–65	13.32 (10.50–16.73)	0.23	0.18	0.30	<0.001
**Country**	Malaysia	33.58 (32.67–34.50)	1.00			
	Indonesia	33.88 (33.06–34.72)	0.92	0.87	0.97	0.003
	Singapore	31.23 (28.47–34.13)	1.04	0.90	1.20	0.551
	Thailand	42.65 (41.65–43.65)	1.46	1.37	1.55	<0.001
**Income**	Low	36.90 (36.30–37.51)	1.00			
	Middle	34.23 (33.13–35.34)	0.89	0.84	0.95	<0.001
	High	34.25 (32.01–36.56)	0.99	0.89	1.11	0.909

*Omnibus test X^2^ = 1035.36, p < 0.001; Nagelkerke R^2^ = 4.2%; Hosmer & Lemeshow test X^2^ = 36.82, p < 0.001; Classification table 63.8% correct; Multicollinearity checks indicated no multicollinearity between the associated factors; ROC area = 0.605*.

For anxiety, females and non-binary respondents had odds of 1.44 and 1.46, respectively, of having severe or above symptoms of anxiety compared to males. In addition, the odds of meeting this cut-off decreased with age: compared to 18- to 29-year-olds, the odds of symptoms of this severity were 0.55, 0.38, and 0.22 among 30- to 39-year-olds, 40- to 49-year-olds, and 50- to 65-year-olds, respectively. Respondents from Indonesia were 8% more likely than those from Malaysia to experience severe or higher symptoms. Compared to respondents in the low-income category, those in the middle-income and high-income categories were 21 and 23% less likely to report severe or above anxiety symptoms, respectively.

With regards to depression symptoms, female and non-binary respondents had odds of 1.39 and 1.42 of reporting symptoms at or above the severe cut-off compared to males, respectively. Increasing age was associated with decreased odds of severe to extremely severe symptoms. Indeed, compared to 18- to 29-year-olds, 30- to 39-year-olds were 46% less likely to experience symptoms of this severity, while 40- to 49-year-olds and 50- to 65-year-olds were 66 and 80% less likely to experience these symptoms, respectively. Residents of Indonesia were 31% less likely than residents of Malaysia to experience these symptoms, but residents of Singapore were 33% more likely than residents of Malaysia to do so. Middle and high income were both associated with approximately 15% lower odds of experiencing severe or above symptoms compared to low income.

For the stress dimension, females and non-binary respondents were 48 and 42% more likely than males to have severe to extremely severe stress symptoms, respectively. In addition, 30- to 39-year-olds were less than two-thirds as likely as 18- to 29-year-olds to report symptoms meeting the severe cut-off. In addition, the odds of 40- to 49-year-olds and 50- to 65-year-olds experiencing symptoms of this severity compared to the youngest age group were 0.44 and 0.23, respectively. Residents of Indonesia were 8% less likely to experience severe or above stress symptoms compared to residents of Malaysia, while residents of Thailand were 46% more likely to experience these symptoms. Finally, the odds of being above the severe cut-off for middle-income respondents were 0.89 that of low-income respondents.

## Discussion

### Psychological distress 18 months post-pandemic declaration

The current study indicates that there is a high prevalence of psychological distress in a Southeast Asian internet-based sample in October 2021, 18 months after the declaration of COVID-19 as a global pandemic. In our sample, 46.86% experienced severe or above symptoms of depression, 49.34% experienced symptoms of anxiety, and 36.19% experienced symptoms of stress above the severe cut-off. These high prevalences are concerning and highlight a widespread impact of the COVID-19 pandemic on mental health in Southeast Asia, as well as an enduring high point prevalence of psychological distress. While we anticipated findings consistent with previous studies identifying elevated mental health problems since the beginning of the pandemic (26) and high rates of negative psychological symptoms among internet users (11), the magnitude of the psychological distress identified in our study is alarming.

### Sociodemographic differences in mental health

Our study also showed that this impact of the pandemic on the point prevalence of mental severe symptoms of psychological distress 18 months after the pandemic's onset is seen particularly in female and non-binary respondents, as well as younger adults and those from low-income households. Female respondents' odds of experiencing severe or above symptoms of anxiety, depression, and stress compared to men ranged between 1.39 and 1.48. This finding is in line with previous findings on gender differences in psychopathology during the COVID-19 pandemic in Southeast Asia (27, 28). Several factors are likely to have contributed to higher anxiety, depression, and stress in females during the pandemic. Indeed, there are gender differences in stress response systems and females tend to have a greater arousal response to stress (29). In addition, during times of disaster, including disease outbreaks, the burden of productive, reproductive, and community work borne by women tends to increase (30), leading to a deterioration of their wellbeing as they take up greater responsibilities (31). In Singapore, for example, mothers were found to be more likely than fathers to have poor to moderate work-family balance during the pandemic (32), illustrating the unequal impact of the pandemic and social distancing measures. Furthermore, evidence indicates that females are more likely than males to believe in COVID-19 conspiracy theories – including threatening ones, which can lead to anxiety and distress (33) and may have also contributed to the gender difference observed in our study.

Our findings of increased odds of severe psychological symptoms in non-binary respondents are consistent with the high rates of mental health problems in transgender and non-binary individuals documented in other studies (34, 35). The pattern of gender differences in our study, in which the prevalence of psychological distress was lowest among males and highest among non-binary individuals, is also the same as that reported in a recent international, multicenter study (33). Little is known about the prevalence of psychological distress in non-binary people in Southeast Asia, however, and our study is one of the first to identify the prevalence of depression, anxiety, and stress in this population. These findings are crucial as mental health challenges in this group are attributed to a variety of social and structural factors, including stigma, social exclusion, and a lack of social support, that are especially common in several Southeast Asian countries where the gender non-conforming community is highly stigmatized (36). Moreover, the COVID-19 pandemic is likely to have exacerbated the mental health challenges experienced by non-binary individuals as protective factors against psychological problems, including gender-affirming healthcare and social connectedness (34, 37), were less available during the pandemic. Many non-binary and transgender individuals have also reported decreased time living according to their gender during the pandemic, leading to increased symptoms of depression and anxiety (38). The high rates of severe depression, anxiety, and stress symptoms in non-binary individuals highlights the importance of ensuring that non-gender conforming individuals continue to have access to gender-affirming healthcare and social support (38).

Moreover, the current study found that younger adults have been disproportionately affected in all three dimensions of mental health, in line with previous research on emerging adults (18- to 29-year-olds) during the pandemic (28, 39). Previous work has shown that younger adults are more concerned than older adults about the threat of COVID-19 on multiple areas including physical health, mental wellbeing, and financial resources (40). Younger adults were especially vulnerable to mental health problems during the pandemic, as it exacerbated the instability and uncertainty that already characterize the transitional period of emerging adulthood (41). Indeed, a sample of Malaysian university students identified financial constraints and uncertainty about the future as some of the main stressors they faced during the pandemic and lockdown (42). Moreover, 18- to 24-year-olds were disproportionately affected by job loss during the pandemic (43, 44), which can have a significant impact on mental health outcomes including anxiety, depression, and life satisfaction (45). Importantly, young adults are also more likely to be more negatively impacted by the stressful and challenging circumstances created by the pandemic because their coping skills tend to be less developed than those of older adults (46). A study of UK adults reported that, during the pandemic, older adults were less likely to use avoidant coping strategies than younger adults (47), and demonstrated more resilience, a key protective factor against psychological distress (48). In addition, use of negative coping styles was shown to be associated with psychological problems among a sample of Chinese youth during the COVID-19 pandemic (49).

We also identified that high- and middle-income levels were associated with decreased odds of experiencing severe anxiety, depression, and stress symptoms compared to lower income levels. These findings are consistent with existing evidence of a relationship between low socioeconomic status and mood and anxiety disorders (50, 51). This relationship can be explained by the social causation hypothesis, which posits that low income can precipitate mental illness by causing adversity, stress, and a reduced capacity to cope (52). In addition, social support has been shown to moderate the relationship between economic hardship and mental health (53). This is important in the context of the COVID-19 pandemic during which many people lost their social support systems and may help explain the high rate of psychological distress in our sample, as the majority was low-income and may have been especially impacted by the lack of social support in this period of economic difficulty.

### Regional differences in mental health

The prevalence of severe stress symptoms was highest among respondents from Thailand, who had significantly higher odds of stress symptoms than Malaysian respondents. This finding is in line with Wang et al.'s (12) recent study on depression, anxiety, and stress symptoms in seven Asian countries, including Malaysia and Thailand, which also reported the highest stress scores among Thai respondents. Interestingly, however, unlike in Wang et al.'s (12) study, this pattern did not hold for anxiety and depression: in our study, residing in Thailand was associated with non-significantly different odds of depression or anxiety symptoms compared to residing in Malaysia.

The inter-country difference in stress may be associated with differences in the status and economic impact of the COVID-19 pandemic between the countries. While Thailand has the second lowest total reported COVID-19 cases per million (30,389 cases) among the four countries included in the study, after Indonesia [15,404 cases; (54)], the country experienced one of the worst economic downturns in Asia because of the pandemic. Thailand had the largest year-on-year GDP contraction of the four countries included in the study in 2021, at 6.1%, compared to 5.4% in Malaysia (55). Over 70% of Thai households experienced income loss and 23% of Thai respondents in a recent survey reported having lost their job (56), which is associated with increased likelihood of experiencing depressive and/or anxiety symptoms (57). As Thai respondents in our study did not have significantly different odds of anxiety or depression compared to Malaysian respondents, this suggests that the low COVID-19 case count may have had a protective effect on depression and anxiety rates amid these challenging conditions, for example by highlighting the value on human life of the measures contributing to economic uncertainty. Odds of severe/extremely severe stress were nonetheless highest among respondents living in Thailand, indicating that their mental health was not unaffected by the poor economic conditions.

Living in Indonesia was associated with significantly lower odds of experiencing severe symptoms of stress and depression, but significantly higher odds of anxiety symptoms, compared to living in Malaysia. This pattern is interesting and indicates that while the economic and health conditions in Indonesia may be less detrimental to residents' mental health in certain areas, there is some variability in the effect. The lower prevalence of stress and depression in Indonesia may be explained by the country being relatively less affected by the pandemic in terms of year-on-year GDP contraction in 2021 [2.1%; (55)] and reported COVID-19 case numbers, as Indonesia has reported the lowest total case count of the four countries (54). Interestingly, our findings are in spite of Indonesians experiencing a higher level of pandemic-related movement and social restrictions than Malaysians at the time of the study (i.e., October 2021), and these restrictions could explain the higher rates of anxiety among Indonesian respondents. This also suggests that longer-term trends play an important role in shaping mental health, rather than just the current situation. Moreover, at this point in the pandemic when individuals have already experienced strict movement restrictions, the impact of these may not be as stark as early after the declaration of the pandemic, in particular if these measures have been shown to mitigate the health emergency.

Compared to respondents residing in Malaysia, those residing in Singapore had higher odds of depression, but not significantly different odds of anxiety or stress. Singapore had the highest total number of COVID-19 cases per million (48,986) and was the only country with rising daily case numbers in October 2021 (54), which brought about the implementation of stricter social distancing measures at the end of September 2021. In addition, while mobility data from Google (58) indicates that in the months leading up to the period of the study, movement patterns in Malaysia, Indonesia, and Thailand were returning to pre-pandemic levels – albeit still showing differences in some areas – this trend was not reflected in Singapore (Supplementary Figure 1). The elevated odds of depression, but non-significantly different odds of stress and anxiety, in Singapore suggest that the enduring nature of restricted mobility combined with high number of reported COVID-19 cases may be especially conducive to symptoms of depression, by increasing feelings of loneliness and hopelessness, which are both associated with depression (59).

### Deteriorating mental health status in 2021 compared to 2020

As we extrapolate our findings on Southeast Asian adults temporally, our study reveals a higher prevalence of psychological distress 18 months after the declaration of the pandemic compared to the first year of the pandemic (12, 60). Similar to our approach, Wong and colleagues (60) measured the mental health of the Malaysian public cross-sectionally between May and September 2020, using the DASS-21 administered through the internet. Their study revealed a progressive increase in the proportion of respondents experiencing problematic psychological symptoms over the 5-month study period. The highest prevalence of respondents reporting moderate of above symptoms of depression (59.2%), anxiety (55.1%), and stress (30.6%) was in the last month of the study period. One year on from Wong et al.'s study, this upward trend seems to have continued, with our study reporting an even higher prevalence of moderate to extremely severe depression (66.77%), anxiety (66.04%), and stress (50.98%) among Malaysian respondents.

This temporal increase in psychological distress is also apparent when comparing the DASS scores from our study with those reported by Wang et al. (12) in Thailand and Malaysia in the period after COVID-19 became an epidemic in each country. Indeed, for both countries, mean scores for depression, anxiety, and stress were 0.7 to 11.6 points higher in our study. The smallest difference was for the stress score in Thailand and the largest difference was for the depression score in Malaysia. This increase in scores over time is consistent with evidence of a deterioration in mental health in Italian and Spanish samples throughout the pandemic (61, 62) and suggests that individuals in Southeast Asia are experiencing pandemic burnout as a result of the stress associated with the health crisis compounding over time (63). It should be noted, however, that the differences in the prevalence of psychological distress between our study and those conducted earlier in the pandemic could reflect differences in the samples' socio-demographic characteristics, rather than temporal changes. Indeed, our sample included a higher proportion of younger adults and low-income individuals than Wang et al.'s (12) or Wong et al.'s (60), both socio-demographic characteristics associated with symptoms of depression, anxiety, and stress.

Together with previous literature, our findings demonstrate the persistence of the mental health impact of the pandemic on Southeast Asians more than one year after its onset (12, 60). This lingering impact seems to be consistent with what has been observed in previous viral outbreaks, including the 1918–1919 influenza pandemic, the Severe Acute Respiratory Syndrome (SARS) outbreak in 2002, and the Middle East Respiratory Syndrome (MERS) outbreak starting in 2012 (64–66). Indeed, Mamelund (67) described an increase in the number of first-time hospitalizations for influenza-related mental disorders by an annual factor of 7.2 in the 6 years after the 1918 influenza pandemic. While many have attributed the psychological impact of viral outbreaks to stressors during and after quarantine such as fear of infection, frustration and boredom, inadequate supplies or information, finances, and stigma (1), others have emphasized the role of biological factors associated with viral infections, such as inflammation, in contributing to psychological morbidity, including anxiety disorder, insomnia, and dementia (66, 68, 69). These factors may better explain the temporal deterioration in psychological symptoms and longitudinal cohort studies including these biological factors are therefore needed to further examine the progression of the mental health impact of the COVID-19 pandemic over time.

### Strengths and limitations

This study utilized the internet as the medium of dissemination of survey questions. As a result, a large sample was recruited within a month, over a large geographical area, otherwise not feasible with face-to-face recruitment. In addition, by including the residents of four Southeast Asian countries experiencing different socioeconomic conditions and COVID-19-related social restrictions, this study provides insights into how different dimensions of psychological distress are related to these variables.

However, several limitations of this study should be acknowledged when considering its findings. First, this study utilized a wholly internet-based approach and people with no access to the internet were excluded. However, the countries in which this study was conducted have a high proportion of population using the internet: 89.6% in Malaysia, 75.9% in Singapore, 77.8% in Thailand, and 53.7% in Indonesia (70). Second, the self-selected nature of the sample is a possible source of bias. Recruitment materials for the survey highlighted the value of gaining insights into one's own mental health status through participation, and consequently, individuals opting to participate in the study may be more likely than the target population to suspect that they are experiencing psychological distress. This may have led to an over-representation of the prevalence of the psychological symptoms measured in the study. Third, the cross-sectional nature of the study prevents us from ascertaining a cause-effect relationship between the pandemic and respondents' mental health status. Moreover, in measuring mental health status at only one point in time, this study is unable to determine whether or not the elevated point prevalence reflects long-lasting symptoms among the individuals whose mental health was negatively impacted early in the pandemic. Fourth, while there are many factors contributing to mental wellbeing, including ethnicity, education level, the physical environment, and social support networks (71), this study only included four demographic factors (age, gender, country of residence, and income level). The small number of independent variables included in the regression is likely to account for the model's low *R*^2^. Despite the model's low explanatory power, however, the independent variables included in it are significant, which helps identify high-risk populations. Finally, our sample consists of a higher proportion of females (76%) and adults aged 18 to 29 years (74%) than the general population, limiting the representability of our findings.

## Conclusions

Overall, this study provides evidence of the differing impact of the COVID-19 pandemic across demographic groups in Southeast Asia, consistent with global trends. The prevalence of depression, anxiety, and stress symptoms in an Southeast Asian internet-based sample is high 18 months after the declaration of COVID-19 as a global pandemic. Females, non-binary respondents, younger adults, and those from low-income households are more likely to experience severe to extremely severe symptoms in all three dimensions of mental health. Moreover, our findings on the differences in the mental health status of respondents between countries suggest that a complete picture comprising economic conditions, the public health situation, and social and movement restrictions should be considered in order to understand the effects of a disaster such as a pandemic on the mental health of the population. Crucially, comparison of our findings with those of other Southeast Asian studies in the year following the declaration of the pandemic further indicates that the mental health status of this population has deteriorated over time.

## Data availability statement

The raw data supporting the conclusions of this article will be made available by the authors, without undue reservation.

## Ethics statement

The studies involving human participants were reviewed and approved by Sunway Medical Centre Independent Research Ethics Committee. The participants provided their informed consent digitally to participate in this study.

## Author contributions

WT and FM conceptualized the study. WT and JJ acquired the data. JJ performed the statistical analysis with help from KW. JJ, WT, KW, and FM contributed to the interpretation of the results. JJ wrote the original draft of the manuscript with contributions from WT. KW, FM, and TO reviewed and edited the manuscript. All authors approved the final version of the manuscript.

## Funding

This study was funded by Naluri Hidup Sdn Bhd.

## Conflict of interest

Authors WT, JJ, and TO are employed by Naluri Hidup Sdn Bhd. The authors declare that this study received funding from Naluri Hidup Sdn Bhd. The funder had the following involvement in the study: study design, collection, analysis, interpretation of data, the writing of this article and the decision to submit it for publication. The remaining authors declare that the research was conducted in the absence of any commercial or financial relationships that could be construed as a potential conflict of interest.

## Publisher's note

All claims expressed in this article are solely those of the authors and do not necessarily represent those of their affiliated organizations, or those of the publisher, the editors and the reviewers. Any product that may be evaluated in this article, or claim that may be made by its manufacturer, is not guaranteed or endorsed by the publisher.
