# Supplementary material for: Eighteen months into the COVID-19 pandemic: The prevalence of depression, anxiety, and stress symptoms in Southeast Asia and the associated demographic factors
Source: Front Public Health. 2022 Aug 4;10:863323. doi: 10.3389/fpubh.2022.863323 (PMC9387355; doi:10.3389/fpubh.2022.863323)
Supplement: Supplementary file 1 [file Table_1.DOCX]

**Supplementary Table 1:** Results from simple logistic regression analyses on the factors associated with severe/extremely severe anxiety, depression, and stress symptoms

|  |  | **Anxiety** | | | | **Depression** | | | | **Stress** | | | |
| --- | --- | --- | --- | --- | --- | --- | --- | --- | --- | --- | --- | --- | --- |
|  |  | Odds ratio | 95% CI Lower | 95% CI Upper | Sig | Odds ratio | 95% CI Lower | 95% CI Upper | Sig | Odds ratio | 95% CI Lower | 95% CI Upper | Sig |
| Gender | Male | 1.00 |  |  |  | 1.00 |  |  |  | 1.00 |  |  |  |
|  | Female | 1.46 | 1.38 | 1.53 | <0.001 | 1.43 | 1.36 | 1.51 | <0.001 | 1.49 | 1.41 | 1.58 | <0.001 |
|  | Other | 1.53 | 1.22 | 1.92 | <0.001 | 1.74 | 1.39 | 2.18 | <0.001 | 1.84 | 1.46 | 2.31 | <0.001 |
| Age | 18-29 | 1.00 |  |  |  | 1.00 |  |  |  | 1.00 |  |  |  |
|  | 30-39 | 0.53 | 0.50 | 0.56 | <0.001 | 0.57 | 0.54 | 0.60 | <0.001 | 0.63 | 0.60 | 0.67 | <0.001 |
|  | 40-49 | 0.35 | 0.31 | 0.38 | <0.001 | 0.36 | 0.33 | 0.40 | <0.001 | 0.44 | 0.39 | 0.49 | <0.001 |
|  | 50-65 | 0.19 | 0.15 | 0.24 | <0.001 | 0.21 | 0.17 | 0.26 | <0.001 | 0.23 | 0.18 | 0.30 | <0.001 |
| Country | Malaysia | 1.00 |  |  |  | 1.00 |  |  |  | 1.00 |  |  |  |
|  | Indonesia | 1.25 | 1.18 | 1.31 | <0.001 | 0.81 | 0.77 | 0.85 | <0.001 | 1.01 | 0.96 | 1.07 | 0.627 |
|  | Singapore | 0.89 | 0.79 | 1.01 | 0.081 | 1.09 | 0.96 | 1.23 | 0.191 | 0.90 | 0.78 | 1.03 | 0.122 |
|  | Thailand | 0.97 | 0.92 | 1.02 | 0.267 | 1.07 | 1.01 | 1.13 | 0.023 | 1.47 | 1.39 | 1.56 | <0.001 |
| Income | Low | 1.00 |  |  |  | 1.00 |  |  |  | 1.00 |  |  |  |
|  | Middle | 0.65 | 0.62 | 0.69 | <0.001 | 0.80 | 0.76 | 0.84 | <0.001 | 0.89 | 0.84 | 0.94 | <0.001 |
|  | High | 0.57 | 0.51 | 0.63 | <0.001 | 0.70 | 0.63 | 0.77 | <0.001 | 0.89 | 0.80 | 0.99 | 0.028 |
