# Supplementary material for: Eighteen months into the COVID-19 pandemic: The prevalence of depression, anxiety, and stress symptoms in Southeast Asia and the associated demographic factors
Source: Front Public Health. 2022 Aug 4;10:863323. doi: 10.3389/fpubh.2022.863323 (PMC9387355; doi:10.3389/fpubh.2022.863323)

Supplementary Figure 1: 7-day rolling average Google mobility data in Malaysia, Indonesia, Singapore, and Thailand for the 6 months preceding 31st October 2021

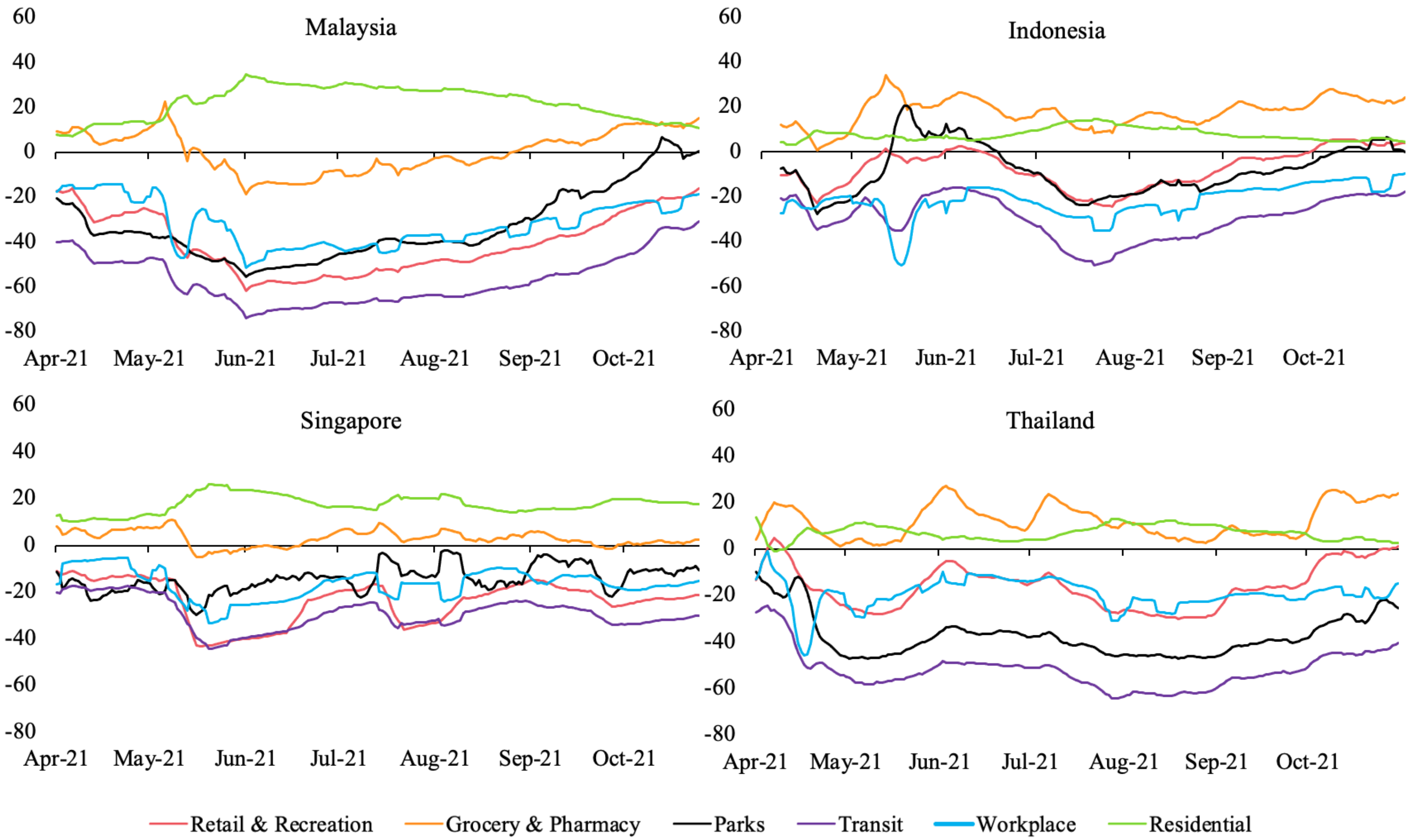

Supplement: Supplementary file 2 [file Image_1.PDF]
